# Preparation of sub-microspherical Fe_3_O_4_@PDA-Pd NPs catalyst and application in catalytic hydroreduction reaction of halogenated aromatic nitro compounds to prepare halogenated aromatic amines

**DOI:** 10.1186/s13065-019-0649-9

**Published:** 2019-11-13

**Authors:** Haichang Guo, Renhua Zheng, Huajiang Jiang, Zhenyuan Xu, Aibao Xia

**Affiliations:** 1 School of Pharmaceutical and Material Engineering, Taizhou University, Taizhou, 318000 China; 2 Zhejiang Key Laboratory of Green Pesticides and Cleaner Production Technology, Catalytic Hydrogenation Research Center, Zhejiang University of Technology, Hangzhou, 310014 China

**Keywords:** Catalytic hydrogenation, Dopamine, Fe_3_O_4_ sub-microsphere nano-palladium, Halogenated aromatic, Nano-palladium

## Abstract

**Background:**

The side reactions of dehalogenation or C–N coupling tend to occur when halogenated aromatic amines are prepared by catalytic hydrogenation reduction of halogenated aromatic nitro compounds. In this paper, we prepared the sub-microspherical Fe_3_O_4_@PDA-Pd NPs catalyst apply it efficiently in the hydrogenation reduction of halogenated aromatic nitro compounds to prepare the halogenated aromatic amines under atmospheric pressure. The catalyst shows a high selectivity of greater than 96% and can effectively inhibit the occurrence of the side reactions of dehalogenation and C–N coupling.

**Results:**

The optimum condition of the hydroreduction reaction is when tetrahydrofuran is used as solvent and the reaction happens at 50 °C for 5 h. The selectivity of the chlorinated aromatic amine and the fluorinated aromatic amine products exceed 99% and the yield exceeds 90%. Only a small amount of dehalogenated products and C–N coupling by-products were produced in the brominated aromatic compound and the iodinated aromatic compound.

**Conclusion:**

We developed a promising method for preparing the superparamagnetic and strongly magnetic Fe_3_O_4_@PDA core–shell sub-microsphere-supported nano-palladium catalyst for catalyzing the hydrogenation reduction of halogenated aromatic nitro compounds. The halogenated aromatic amines were efficiently and highly selectively prepared under atmospheric pressure, with the side reactions of dehalogenation and C–N coupling effectively inhabited simultaneously.

## Introduction

Aniline compounds are important intermediates in organic synthesis and are widely used in medicines [1], additives [2], flame retardants [3], dyes and surfactants [4]. Reducing aromatic nitro compounds is the most important and simplest method for preparing the aniline compounds. And in industry, there are several major ways to prepare the aniline compounds such as catalytic hydrogenation, hydrazine hydrate, active metal and sulfide reduction. In comparison, the latter three were gradually eliminated due to their toxicity, harmfulness and sewage pollution, and only the catalytic hydrogenation gradually prevail due to its clean reaction process [5–8]. Halogenated aromatic amines are important classifications of aniline compounds especially in pesticides, such as, *p*-chloroaniline used to prepare Monolinuron [9], *m*-chloroaniline used to prepare Barban [10], and 3-chloro-4-methylaniline used to prepare Chlorotoluron [11]. However, when the halogenated aromatic amines are prepared by catalytic hydrogenation reduction of halogenated aromatic nitro compounds, the side reactions of dehalogenation [12] or C–N coupling [13] are easy to occur. Therefore, using improved highly efficient and selective catalytic hydrogenation to prepare the halogenated aromatic amines becomes a key technique to prevent the dehalogenation and C–N coupling side reactions. Recently, some useful results had been achieved by using gold complexes, palladium complexes and platinum complexes as catalysts in this reduction reaction [14–17].

**Fig. 1:**
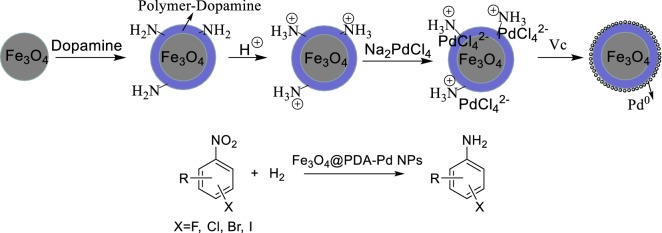
Preparation of Fe_3_O_4_@PDA-Pd NPs and its catalysis in hydrogenation reaction of halogenated aromatic nitro compounds

Palladium is used to catalyze the hydrogenation of many unsaturated compounds such as olefins [18], alkynes [19], nitro compounds [20], carbonyl compounds [21] and nitriles [22], as well as to catalyze the dehalogenation, debenzylation, Suzuki–Miyaura coupling, Heck and Sonogashira reactions [23–25]. It is known that nano-palladium particles (Pd NPs) supported on the Fe_3_O_4_ particles can improve both the catalytic performance of palladium and the selectivity of the catalytic reactions; also, the separation and recycling of the catalyst is very simple [26–31]. In this paper, we prepared the sub-micro-spherical Fe_3_O_4_@PDA-Pd NPs complex as a high performance catalyst. The preparation procedures include: first, the surface of the Fe_3_O_4_ particles are covered by polydopamine (PDA) layer through the dopamine autoagglutination to form the Fe_3_O_4_@PDA core–shell structures. Then the amino group of the sub-microspheres are combined with proton through protonation with positive electricity. The PdCl_4_^2−^ ions are then dispersed on the Fe_3_O_4_@PDA core–shell surface by charge attraction. And the nano-palladium supported on the sub-microspheres is further prepared by reduction to form the Fe_3_O_4_@PDA-Pd NPs complex. This complex catalyst is used to catalyze the hydrogenation reduction of a halogenated aromatic nitro compound to produce the halogenated aromatic amine (Fig. 1). The conversion and selectivity of the reaction are both very high, and the occurrence of the dehalogenation and C–N coupling side reactions are effectively suppressed at the same time.

## Results and discussion

**Fig. 2:**
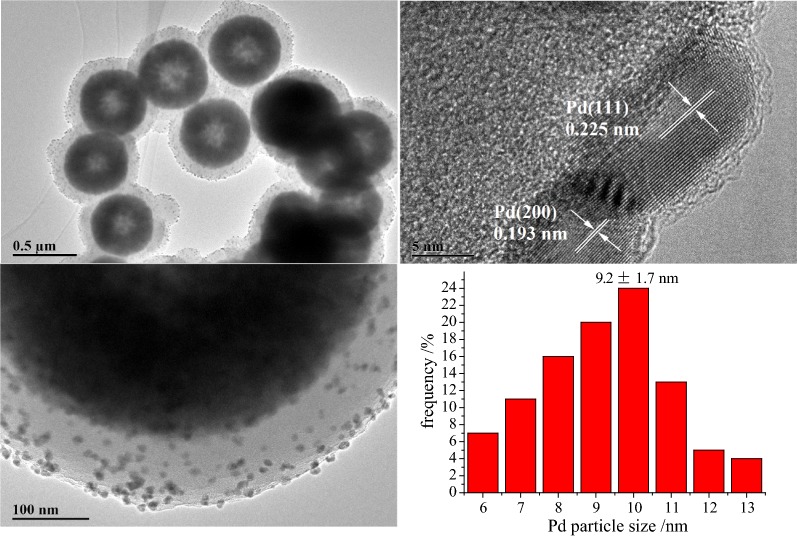
TEM images of Fe_3_O_4_@PDA@Pd and Pd particle size

The prepared Fe_3_O_4_@PDA-Pd NPs catalyst was characterized by the transmission electron microscopy (TEM) observation and magnetic testing. The TEM image (Fig. 2) shows that the Fe_3_O_4_@PDA-Pd NPs catalyst presents core–shell micro structures which are centered on the Fe_3_O_4_ sub-microspheres. The dopamine layer is uniformly coated on the surface of the Fe_3_O_4_ sub-microspheres for form the shell-like dopamine with thickness distributed in the range of 80–90 nm. The nano palladium particles, with diameters ranging from 7 to 12 nm and average diameter of 9.2 nm, are dispersed on the dopamine shell.

**Fig. 3:**
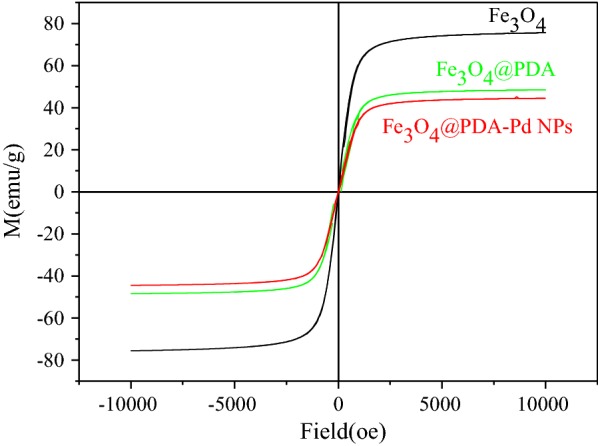
Magnetization curves of Fe_3_O_4_, Fe_3_O_4_@PDA and Fe_3_O_4_@PDA-Pd NPs

Figure 3 shows the magnetization curves of the Fe_3_O_4_, Fe_3_O_4_@PDA and the Fe_3_O_4_@PDA-Pd NPs sub-microspheres under room temperature (300 K). It can be seen that the maximum saturation magnetic field strengths of the three kinds of sub-microspheres are 75, 48 and 45 emu/g, respectively, and their coercivity is 0. The presence of the PDA layer reduced the maximum saturation value of the magnetic field strength of the Fe_3_O_4_@PDA-Pd NPs sub-microspheres, but the Fe_3_O_4_@PDA-Pd NPs sub-microspheres still have superparamagnetic and strongly magnetic properties. Therefore, the Fe_3_O_4_@PDA-Pd NPs sub-microspheres can be easily dispersed into and then separated from the reaction system.

We then used the prepared Fe_3_O_4_@PDA-Pd NPs sub-microspheres as catalyst for the halogenated aromatic amines preparation through hydrogenation of the halogenated aromatic nitro compounds. In order to investigate the exact role which the Fe_3_O_4_@PDA-Pd NPs played in the catalytic hydrogenation reduction of the halogenated aromatic nitro compounds and its effect on inhibiting the side reaction of dehalogenation, the reaction temperature, solvent and reaction time were optimized under atmospheric pressure using *p*-nitrochlorobenzene as the substrate (Table 1). The results show that when ethanol is used as the solvent, the hydrogenation reaction rate is the fastest (Table 1, entry 1); but due to the alkylation reaction of ethanol and *p*-chloroaniline to form about 4% of *N*-ethyl-*p*-chloroaniline, the selectivity of *p*-chloroaniline (I) is lowered. The *N*-ethyl-*p*-chloroaniline was determined by GC–MS. Then when tetrahydrofuran is used as the reaction solvent (Table 1, entry 2), the reaction rate becomes slow; but the selectivity to *p*-chloroaniline (I) becomes higher, and the by-product of dechlorination (II) becomes rare. The reaction rate increases with increase in the reaction temperature, and the conversion rate of *p*-nitrochlorobenzene and the selectivity to *p*-chloroaniline (I) are both greater than 99% at 50 °C (Table 1, entry 3). The selectivity of *p*-chloroaniline (I) with Fe_3_O_4_@PDA-Pd NPs as catalyst is much higher than that with Pd/C as catalyst (Table 1, entry 4). Under the same conditions, about 13% of the products dechlorinated with the latter as catalyst. Therefore, the optimum condition of the hydroreduction reaction is to use tetrahydrofuran as solvent and keep the reaction at 50 °C for a reaction time of 5 h (Table 1, entry 3).

**Table 1:** Fe_3_O_4_@PDA-Pd NPs-catalyzed hydroreduction reaction of *p*-nitrochlorobenzene
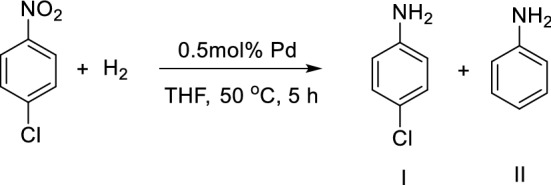

Entry	Catalyst	Solvent	Temperature, time	Conversion^a^/%	Selectivity (I)^a^/%
1	Fe_3_O_4_@PDA-Pd NPs	EtOH	30 °C, 4 h	>99	95^b^
2	Fe_3_O_4_@PDA-Pd NPs	THF	30 °C, 4 h	81	98
**3**	Fe_3_O_4_@PDA-Pd NPs	*THF*	*50* *°C, 5* *h*	*>* *99*	*>* *99*
4	Pd/C	THF	50 °C, 2.5 h	> 99	84^c^

^a^GC; ^b^ about 4% *N*-ethyl-*p*-chloroaniline in the product; ^c^ about 13% aniline in the product

The extent of the reaction under the optimal reaction conditions (Table 1, entry 3) were examined and the results are shown in Table 2. It can be seen that the Fe_3_O_4_@PDA-Pd NPs catalyst has high selectivity and high yield for the hydroreduction of halogenated aromatic nitro compounds in preparing the halogenated aromatic amines. The halogenated aromatic amine has a selectivity of more than 96% and a yield of over 84%. In particular, the selectivity of the chlorinated aromatic amine and the fluorinated aromatic amine exceeds 99%, and the yield exceeds 90%. There is no C–N coupling reaction in the fluorinated aromatic compounds happened (Table 2, entry 8, 9, 12), and the dechlorination of chlorinated aromatic compounds (Table 2, entry 1–5, 13) is rare. Only a small amount of dehalogenated products and C–N coupling by-products was produced in the brominated aromatic compound (Table 2, entry 7) and the iodinated aromatic compound (Table 2, entry 6).

**Table 2:**
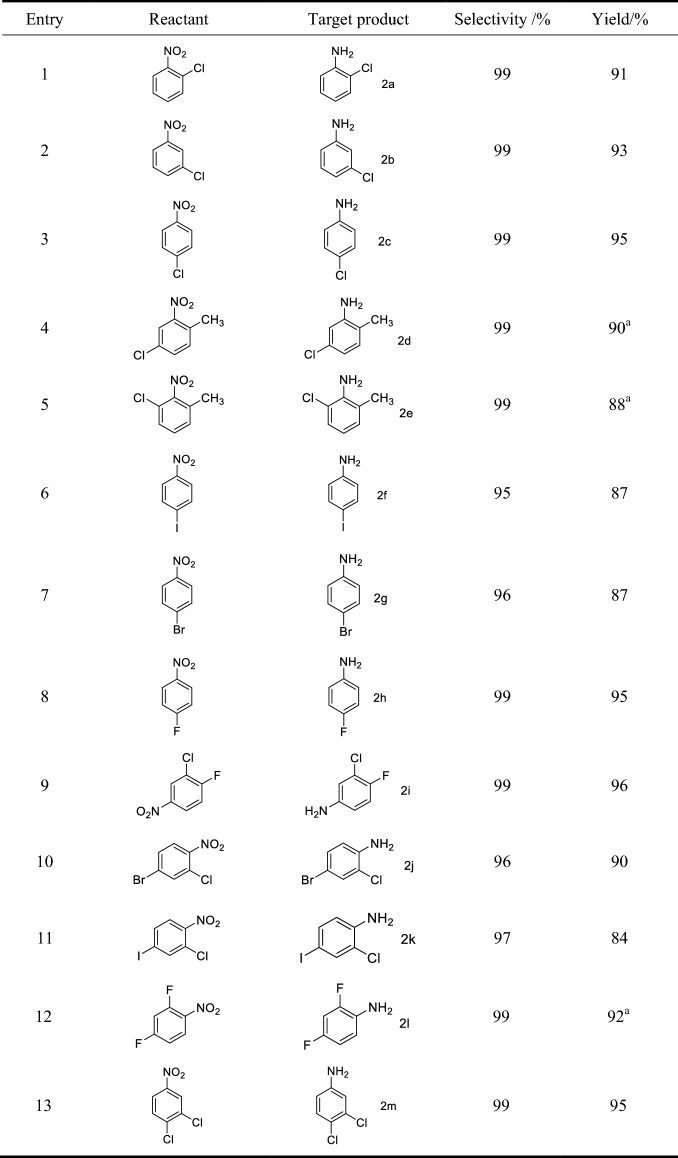
Fe_3_O_4_@PDA-Pd NPs-catalyzed hydrogenation reaction of halogenated aromatic nitro compounds
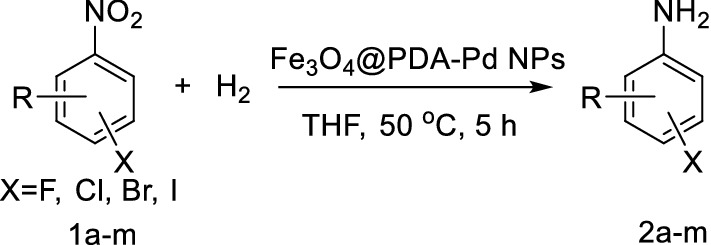

^a^7 h

## Experimental

### General

All reagents used in the experiment are commercially available without further purification. The transmission electron microscopy (TEM) image was obtained on a JEOL JEM-2100F field transmission electron microscope. The magnetic property information of the Fe_3_O_4_@PDA-Pd NPs was obtained on a Quantum Design DynaCool-9 vibrating sample magnetometer. And the ^1^H NMR and ^13^C NMR spectra were recorded on a Bruker Avance 400 MHz spectrometer using tetramethylsilane (TMS) as internal standards.

The Fe_3_O_4_ particles were prepared according to the method specified in Ref. [32].

### General procedure for the preparation of the Fe_3_O_4_ @PDA

About 0.15 g of strong aqueous ammonia was dissolved in 50 mL of deionized water. Then, 0.1 g of Fe_3_O_4_ and 0.16 g of dopamine hydrochloride were added into the solution, and the mixture was placed under uniform ultrasonic dispersion and mechanically stirred at 40 °C for 24 h. After the reaction is completed, the solid and the liquid are separated by a magnet. The solid product was then placed under ultrasonic washing using 25 mL * 3 deionized water and 25 mL * 3 ethanol, and the washed solid was used directly in the next step.

### General method for the preparation of the Fe_3_O_4_ @PDA-Pd NPs

The Fe_3_O_4_@PDA was taken and placed under ultrasonic washing using 25 mL of deionized water, 0.1 N of 25 mL hydrochloric acid, 25 mL of deionized water, and 25 mL of ethanol. Approximately 40 mL of ethanol and 4 mL of deionized water were added into the mixture, which was then mechanically stirred at 10 °C. Subsequently, about 0.3 mL NaPdCl_4_ aqueous solution (palladium content 2.5 mg) was slowly added into the mixture dropwise, and then the mixture was continuously stirred for another 3 h. A solution containing 60 mg of ascorbic acid and 6 mL of deionized water was slowly added into the mixture in 20 min. Then, the reaction was continued for another 2 h. The solid and liquid were separated by a magnet. The reaction product was placed under ultrasonic washing using 25 mL of ethanol, 25 mL * 3 of deionized water and 25 mL * 3 of ethanol. The solid was stored in 25 mL of ethanol and sealed with nitrogen (about 0.1 g after drying).

### General procedure for the hydrogenation of halogenated aromatic nitro compounds to prepare halogenated aromatic amines 2a–m

Approximately 0.1 g of Fe_3_O_4_@PDA-Pd NPs catalyst was used. The aforementioned solid and liquor were separated using a magnet and placed under ultrasonic washing using 10 mL * 3 THF. Then, 10 mL THF and 4.3 mmol halogenated aromatic nitro compounds were added. Nitrogen and hydrogen were introduced alternatively. The magnetic stirring was carried out at 50 °C. Hydrogen (hydrogen balloon) was introduced into the reaction at atmospheric pressure for 4–6 h. At the end of the reaction, the catalyst was separated and recovered by a magnet, and the product was separated by column chromatography (*n*-hexane/dichloromethane) after the reaction liquid was concentrated.

#### 2-Chloroaniline (2a)

^1^H NMR (400 MHz, CDCl_3_) δ 7.23 (dd, *J* = 8.0, 1.1 Hz, 1H), 7.05 (td, *J* = 8.0, 1.3 Hz, 1H), 6.74 (dt, *J* = 8.8, 4.4 Hz, 1H), 6.68 (td, *J* = 7.8, 1.4 Hz, 1H), 4.02 (s, 2H). ^13^C NMR (101 MHz, CDCl_3_) δ 142.92 (s), 129.44 (s), 127.66 (s), 119.31 (s), 119.05 (s), 115.90 (s).

#### 3-Chloroaniline (2b)

^1^H NMR (400 MHz, CDCl_3_) δ 7.05 (dd, *J* = 10.4, 5.6 Hz, 1H), 6.71 (ddd, *J* = 7.9, 1.8, 0.7 Hz, 1H), 6.65 (t, *J* = 2.1 Hz, 1H), 6.53 (ddd, *J* = 8.1, 2.2, 0.6 Hz, 1H), 4.17–3.03 (s, 2H). ^13^C NMR (101 MHz, CDCl_3_) δ 147.66 (s), 134.85 (s), 130.36 (s), 118.48 (s), 114.95 (s), 113.23 (s).

#### 4-Chloroaniline (2c)

^1^H NMR (400 MHz, CDCl_3_) δ 7.09 (d, J = 8.7 Hz, 2H), 6.59 (d, J = 8.7 Hz, 2H), 3.56 (s, 2H). ^13^C NMR (101 MHz, CDCl_3_) δ 144.99 (s), 129.14 (s), 123.13 (s), 116.27 (s).

#### 5-Chloro-2-methylaniline (2d)

^1^H NMR (400 MHz, CDCl_3_) δ 6.94 (d, *J* = 8.4 Hz, 1H), 6.65 (d, *J* = 5.5 Hz, 2H), 3.65 (s, 2H), 2.10 (s, 3H). ^13^C NMR (101 MHz, CDCl_3_) δ 145.72 (s), 132.06 (s), 131.34 (s), 120.59 (s), 118.23 (s), 114.48 (s), 16.87 (s).

#### 6-Chloro-2-methylaniline (2e)

^1^H NMR (400 MHz, CDCl_3_) δ 7.12 (d, *J* = 8.0 Hz, 1H), 6.94 (dd, *J* = 7.5, 0.5 Hz, 1H), 6.61 (t, *J* = 7.7 Hz, 1H), 3.97 (s, 2H), 2.18 (s, 3H). ^13^C NMR (101 MHz, CDCl_3_) δ 141.18 (s), 128.72 (s), 127.07 (s), 123.56 (s), 119.15 (s), 118.32 (s), 17.98 (s).

#### 4-Iodoaniline (2f)

^1^H NMR (400 MHz, CDCl_3_) δ 7.40 (d, J = 8.7 Hz, 2H), 6.46 (d, J = 8.7 Hz, 2H), 3.97–3.33 (s, 2H). ^13^C NMR (101 MHz, CDCl_3_) δ 146.08 (s), 137.92 (s), 117.32 (s), 79.41 (s).

#### 4-Bromoaniline (2 g)

^1^H NMR (400 MHz, CDCl_3_) δ 7.22 (d, J = 8.7 Hz, 2H), 6.56 (d, J = 8.7 Hz, 2H), 3.70 (s, 2H). ^13^C NMR (101 MHz, CDCl_3_) δ 145.50 (s), 131.99 (s), 116.71 (s), 110.10 (s).

#### 4-Fluoroaniline (2 h)

^1^H NMR (400 MHz, CDCl_3_) δ 6.95–6.78 (m, 2H), 6.72–6.50 (m, 2H), 3.48 (s, 2H). ^13^C NMR (101 MHz, CDCl_3_) δ 157.60 (s), 155.26 (s), 142.43 (d), 115.80 (m).

#### 4-Fluoro-3-chloroaniline (2i)

^1^H NMR (400 MHz, CDCl_3_) δ 6.91 (t, *J* = 8.8 Hz, 1H), 6.69 (dd, *J* = 6.1, 2.8 Hz, 1H), 6.60–6.40 (m, 1H), 3.59 (s, 2H). ^13^C NMR (101 MHz, CDCl_3_) δ 152.79 (s), 150.42 (s), 143.16 (d), 120.94 (d), 116.75 (m), 114.28 (d).

#### 4-Bromo-2-chloroaniline (2j)

^1^H NMR (400 MHz, CDCl_3_) δ 7.37 (d, *J* = 2.2 Hz, 1H), 7.15 (dd, *J* = 8.5, 2.2 Hz, 1H), 6.69–6.57 (m, 2H), 4.04 (s, 2H). ^13^C NMR (101 MHz, CDCl_3_) δ 142.11 (s), 131.63 (s), 130.54 (s), 119.93 (s), 116.87 (s), 109.36 (s).

#### 2-Chloro-4-iodoaniline (2k)

^1^H NMR (400 MHz, CDCl_3_) δ 7.53 (d, *J* = 2.0 Hz, 1H), 7.31 (dd, *J* = 8.4, 2.0 Hz, 1H), 6.52 (d, *J* = 8.4 Hz, 1H), 4.05 (s, 2H). ^13^C NMR (101 MHz, CDCl_3_) δ 142.73 (s), 137.18 (s), 136.33 (s), 120.23 (s), 117.43 (s), 77.97 (s).

#### 2,4-Difluoroaniline (2l)

^1^H NMR (400 MHz, CDCl_3_) δ 6.82–6.73 (m, 1H), 6.73–6.64 (m, 2H), 3.46 (s, 2H). ^13^C NMR (101 MHz, CDCl_3_) δ 156.49 (d), 154.12 (d), 152.22 (d), 149.82 (d), 130.68 (dd), 116.88 (dd), 110.88 (dd), 103.79 (dd).

#### 3,4-Dichloroaniline (2m)

^1^H NMR (400 MHz, CDCl_3_) δ 7.17 (d, *J* = 8.6 Hz, 1H), 6.75 (d, *J* = 2.7 Hz, 1H), 6.50 (dd, *J* = 8.6, 2.7 Hz, 1H), 3.72 (s, 2H). ^13^C NMR (101 MHz, CDCl_3_) δ 146.02 (s), 132.67 (s), 130.73 (s), 121.08 (s), 116.41 (s), 114.62 (s).

## Conclusions

We developed a method for preparing superparamagnetic and strongly magnetic Fe_3_O_4_@PDA core–shell sub-microsphere-supported nano-palladium catalyst, i.e. Fe_3_O_4_@PDA-Pd NPs. The catalyst was characterized and successfully catalyzed the hydrogenation reduction of halogenated aromatic nitro compounds. The halogenated aromatic amines were efficiently and selectively prepared under atmospheric pressure, which could effectively inhibit the occurrence of the side reactions of dehalogenation and C–N coupling.

## Data Availability

All data and material analyzed or generated during this investigation are included in this manuscript. The raw data can be requested from email of AX: xiaaibao@zjut.edu.cn.

## Abbreviations

PDA: polydopamine
Pd NPs: nano-palladium particles
THF: tetrahydrofuran
TEM: the transmission electron microscopy
